# Surveying the Digital Cytology Workflow in Italy: An Initial Report on AI Integration Across Key Professional Roles

**DOI:** 10.3390/healthcare13080903

**Published:** 2025-04-14

**Authors:** Daniele Giansanti, Elisabetta Carico, Andrea Lastrucci, Enrico Giarnieri

**Affiliations:** 1 Centre TISP, Istituto Superiore di Sanità, 00161 Roma, Italy; 2 Department of Clinical and Molecular Medicine, Cytopathology unit Sapienza University, Sant’Andrea Hospital, 00189 Roma, Italy; elisabetta.carico@uniroma1.it (E.C.); enrico.giarnieri@uniroma1.it (E.G.); 3 Department of Allied Health Professions, Azienda Ospedaliero-Universitaria Careggi, 50134 Florence, Italy; andrea.lastrucci@unifi.it

**Keywords:** digital cytology, artificial intelligence, questionnaires, surveys

## Abstract

Background: The integration of artificial intelligence (AI) in healthcare, particularly in digital cytology, has the potential to enhance diagnostic accuracy and workflow efficiency. However, AI adoption remains limited due to technological and human-related barriers. Understanding the perceptions and experiences of healthcare professionals is essential for overcoming these challenges and facilitating effective AI implementation. Objectives: This study aimed to assess AI integration in digital cytology workflows by evaluating professionals’ perspectives on its benefits, challenges, and requirements for successful adoption. Methods: A survey was conducted among 150 professionals working in public and private healthcare settings in Italy, including laboratory technicians (35%), medical doctors (25%), biologists (20%), and specialists in diagnostic technical sciences (20%). Data were collected through a structured Computer-Assisted Web Interview (CAWI) and a Virtual Focus Group (VFG) to capture quantitative and qualitative insights on AI familiarity, perceived advantages, and barriers to adoption. Results: The findings indicated varying levels of AI familiarity among professionals. While many recognized AI’s potential to improve diagnostic accuracy and streamline workflows, concerns were raised regarding resistance to change, implementation costs, and doubts about AI reliability. Participants emphasized the need for structured training and continuous support to facilitate AI adoption in digital cytology. Conclusions: Addressing barriers such as resistance, cost, and trust is essential for the successful integration of AI in digital cytology workflows. Tailored training programs and ongoing professional support can enhance AI adoption, ultimately optimizing diagnostic processes and improving clinical outcomes.

## 1. Introduction

The digital cytology workflow has evolved through several key stages, driven by the advancement and integration of digital health technologies into cytology practices.

### 1.1. Historical Foundations of the Digital Cytology Workflow

Both digital cytology and digital histology (part of the broader digital pathology field) have historical roots in telepathology, which emerged in the late 20th century, laying the foundation for today’s innovations in digital cytology [1]. Initially, telepathology relied on the transmission of static images [2,3]. However, as technology progressed, telepathology evolved into a dynamic, robot-assisted discipline [4]. Robotic telepathology systems allowed pathologists to remotely control microscopes and examine slides in real time, offering rapid consultations and diagnoses regardless of geographical location [5,6]. This advancement was particularly impactful in underserved areas, bridging gaps in healthcare access and promoting international collaboration.

In these early stages, the digital cytology workflow relied heavily on two key components: the digitization of slides and the mechatronic control of microscopes. The digitization process allowed for the exchange of slides, while the mechatronic systems enabled the remote manipulation of microscopes, with the aim facilitating the examination of slides from different locations.

Despite its innovation, telepathology faced challenges due to limited internet speeds, leading to latency issues [7]. Synchronization problems occurred between the microscope carriage and remote commands, and there was an imperfect alignment of essential functions such as focus, zoom, and translation. To address these issues, virtual microscopy was developed as a solution, operating in a deferred-time mode. Virtual microscopy involves scanning slides, storing them online, and remotely controlling the virtual slides, which are commonly referred to as digital slides.

### 1.2. An Advancement in the Digital Cytology Workflow: The Role of Virtual Microscopy [1] as a Transformative Technology in Microscopy

The Digital cytology workflow has evolved significantly, with important differences compared to digital histology, particularly with the integration of virtual microscopy.

Virtual microscopy [1] enables the scanning, storage, and remote analysis of high-resolution digital slides. This process involves scanning slides with digital scanners [8,9,10,11], creating a digital replica that pathologists can examine remotely, and adjusting focus, zoom, and contrast for detailed inspection.

Its main benefit is remote access and collaboration, allowing pathologists to consult on cases in real time, regardless of location [12]. This is especially valuable in underserved areas. Virtual slides also eliminate physical storage needs, improving logistics and saving space. The technology also brings the potential of efficiency and cost savings, allowing multiple pathologists to access slides without repeated transfers [1,12] with important implication and potential in education and training [13,14,15].

While virtual microscopy simplifies histology, it presents challenges in cytology due to the thinner nature of cellular samples, which requires more precise focus adjustments to capture fine details. Additionally, creating digital slides for cytology is more computationally demanding, as these slides require higher resolution to capture the intricate structures of individual cells. This results in greater memory usage and increased storage requirements compared to histology slides, making the process more resource-intensive [1]. Despite the challenges, emerging potentials in the digital cytology workflow are significantly improving accessibility, diagnostic accuracy, and collaboration, positioning it as a cornerstone in modern pathology.

### 1.3. The Integration of Artificial Intelligence into the Digital Cytology Workflow

Another key step in the evolution of the digital cytology workflow is its integration with artificial intelligence (AI).

Artificial intelligence (AI) has the potential to progressively enhance the digital cytology workflow [16,17].

The integration of AI into digital cytology workflows presents significant potential to enhance diagnostic accuracy, improve workflow efficiency, and facilitate collaboration across different areas of pathology. However, the widespread adoption of AI requires ongoing research, validation, and integration strategies to address the existing challenges. A recent overview of reviews highlights, alongside the potential and opportunities, significant challenges to address in order to achieve the concrete integration of the digital cytology workflow, including AI [18,19,20,21,22,23,24,25,26,27,28,29,30,31,32,33,34,35,36,37]. Ciaparrone et al. (2024) [18] highlighted the potential of AI-driven Computer-Aided Diagnosis (CAD) systems for improving the diagnosis of urothelial carcinomas. These systems could significantly enhance diagnostic accuracy and workflow efficiency, but they emphasize the need for rigorous validation, regulatory approval, and comprehensive training for pathologists to successfully implement them. Zhang et al. (2024) [19] focused on AI’s role in the early diagnosis and risk stratification of thyroid cancer, achieved through the analysis of ultrasound images and molecular markers. While promising, the authors note that further clinical validation and development are necessary before AI tools can be expanded into routine clinical practice. In their 2024 study, Caputo et al. [20] examined how advancements in molecular diagnostics, when integrated with digital pathology, can refine cancer risk assessment strategies. Although such innovations contribute to more accurate diagnoses and reduced healthcare costs, they also highlight the pressing challenge of embedding AI and digital systems effectively within routine clinical operations. Furthermore, they suggested exploring the perspectives of pathologists regarding these new tools. Giovanella et al. (2024) [21] explored the combined use of ultrasound, FNAC, molecular imaging, and AI in refining thyroid nodule diagnosis and reducing unnecessary procedures. They recommended further research to establish the clinical value of AI and its effectiveness in combination with other diagnostic tools. Kim et al. (2024) [22,23] provide a detailed review of integrating AI into digital cytology workflows. While AI has the potential to improve diagnostic accuracy and efficiency, challenges such as technology costs, workflow integration, and the need for standardized protocols must be addressed to ensure its successful implementation. Malik and Zaheer (2024) [24] examined how integrating AI tools like ChatGPT with digital slide analysis can support cancer diagnostics by facilitating data interpretation and knowledge synthesis. Nevertheless, they highlight key obstacles, including system compatibility, algorithmic bias, and unresolved legal considerations. Slabaugh et al. (2023) [25] investigated how Machine Learning (ML) and Deep Learning (DL) techniques are being applied to improve diagnostic accuracy in thyroid cytology and histopathology, aiming to overcome traditional limitations. Despite this progress, the study underscores the necessity for prospective validation, more transparent models, and smoother integration into everyday clinical routines. Focusing on thyroid neoplasms, Lebrun and Salmon (2024) [26] reported recent updates in molecular testing and classification systems, which contribute to more accurate diagnoses and stratifications of risk. Yet, challenges persist, particularly in managing low-risk lesions and embedding AI technologies into existing diagnostic protocols.

Singla et al. (2024) [27] drew attention to AI’s promising role in the identification and classification of fungal infections, leading to improved diagnostic accuracy and faster detection. However, they point out that more extensive studies are needed to fully harness AI’s potential in fungal cytology. Sunny et al. (2023) [28] presented a framework that combines AI with biomarker profiling to enhance the sensitivity and specificity of diagnostics for oral potentially malignant disorders. The authors stress the importance of refining biomarker sets and advancing automation in image analysis, particularly for point-of-care applications. Wong et al. (2023) [29] addressed how ML techniques may advance the diagnostic process in thyroid cytopathology, improving both accuracy and efficiency. Still, they emphasize the importance of using large, heterogeneous datasets and conducting further validation to ensure robust clinical implementation. Ludwig et al. (2023) [30] proposed that AI may assist in better classifying and managing thyroid nodules, potentially minimizing unnecessary interventions. Despite this, further research is required to validate these systems and increase their relevance in clinical settings. Marletta et al. (2023) [31] discussed AI’s capacity to support microbiological diagnostics, particularly through the analysis of cytological images in low-resource environments. To broaden its application, improvements in both technology and data availability are necessary. Tessler et al. (2022) [32] illustrated how AI can aid in assessing thyroid nodules, offering benefits such as greater diagnostic precision and operational efficiency, particularly for less experienced clinicians. Nonetheless, concerns remain around cost, usability, and implementation feasibility. Thakur et al. (2022) [33] explored AI’s contributions to diagnosing non-gynecological cancers, noting its encouraging performance across different tumor types. They recommend expanding annotated datasets and conducting external validation to bolster the clinical translation of AI models. Alrafiah et al. (2022) [34] reviewed the evolution of AI in cytopathology, noting gains in accuracy and workflow optimization. However, they advocate for transparent model development, rigorous validation procedures, and practical clinical integration. Vaikus et al. (2023) [35] highlighted how merging AI with consensus-driven rule systems may help reduce inter-observer variability and promote diagnostic consistency. However, they point out that biases and variability in AI systems need to be addressed for AI’s full potential in cytopathology to be realized. Jorda et al. (2024) [36] focused on AI’s ability to enhance urinary tract cytopathology, improving diagnostic accuracy and patient management. The authors highlight the need for improved alignment between cytology results and tissue samples to reduce the likelihood of false-positive or false-negative findings. Torres et al. (2024) [37] explored the synergy between AI, Fine Needle Aspiration (FNA), and molecular diagnostics in thyroid cytology, leading to enhanced diagnostic precision and better risk assessment. They underline the difficulties of merging AI with conventional diagnostic approaches and stress the importance of thorough validation within real-world clinical environments.

### 1.4. Collaborative Approaches to AI Integration in Digital Cytology: Identifying Challenges, Enablers, and the Path to Effective Implementation—Rationale for the Study

The integration of AI into the digital cytology workflow represents a transformative opportunity for enhancing diagnostic accuracy and efficiency. However, this process requires a highly collaborative approach, engaging various professionals within the diagnostic ecosystem to ensure its success. Biomedical laboratory technicians [38] play a critical role by preparing, staining, and digitizing samples, ensuring that high-quality digital slides are available for AI analysis and expert review. Their work is essential in maintaining the clarity and consistency of the images, which directly impacts the performance of AI tools. Physicians and biologists [39], as primary decision-makers, remain crucial in the final diagnosis. While AI assists by flagging areas of interest, the interpretation of results is still reliant on their clinical expertise, patient history, and the contextual factors surrounding each case. AI currently serves as a supportive tool rather than a replacement for medical professionals, emphasizing the need for ongoing human oversight in diagnostic decision-making. Technical specialists in diagnostic sciences [40] are tasked with the critical role of maintaining and optimizing AI systems, ensuring that they are seamlessly integrated into the existing workflows of clinical practice. They are also responsible for interpreting AI-generated outputs, ensuring that the technology functions effectively and accurately within the diagnostic context.

Despite the promising potential of AI in the digital cytology workflow, its adoption remains in the early stages. Many diagnostic settings have yet to incorporate AI into routine practice, and there are significant hurdles to overcome, such as technical barriers, workflow integration, and training gaps. Identifying and addressing these challenges is essential to facilitate the effective and widespread adoption of AI in clinical settings.

Therefore, this study aims to explore the practical challenges and enablers of AI integration into digital cytology workflows. This study aims to examine the challenges and enablers associated with integrating AI into digital cytology workflows. Through a virtual focus group and structured survey involving key professionals, this study seeks to identify the real-world obstacles and practical needs that must be addressed for its successful integration. The findings will provide stakeholders, including healthcare providers, administrators, and policymakers, with valuable insights to inform decisions on how to align AI integration with the digital cytology workflow. By identifying key areas for improvement, this study will support the development of strategies that promote effective implementation, optimize workflows, and ensure the smooth adoption of AI technologies in the digital cytology workflow.

## 2. Methods

### 2.1. Study Design, Setting, and Participants

This study, conducted from 31 October 2024 to 31 January 2025, aimed to explore the integration of digital cytology and artificial intelligence (AI) in diagnostic workflows within healthcare settings in Italy. This research was conducted using a combination of two primary methods: a Virtual Focus Group (VFG) and a Computer-Assisted Web Interviewing (CAWI) survey. These methods were integrated, with the VFG component embedded within the CAWI platform. This integration enabled the electronic collection of both closed- and open-ended focused responses in a single, efficient process, without requiring physical interaction among participants.

#### 2.1.1. The Integrated Approach: VFG Embedded Within CAWI

The embedding of the VFG component within the CAWI platform offered several distinct advantages:

1. Efficiency in Data Collection:

By integrating the VFG within the CAWI platform, this study combined both structured, quantitative data (via closed-ended questions) and qualitative insights (via open-ended questions) into one seamless process. This allowed for a holistic understanding of the participants’ experiences and opinions on digital cytology and AI tools, all collected electronically in one go.

2. Enhanced Participant Convenience:

The CAWI platform allowed participants to respond to both closed- and open-ended questions at their own convenience, from any location with internet access. This flexibility encouraged more thoughtful responses and eliminated the pressure of real-time, physical group discussions.

3. Asynchronous Data Collection:

The VFG component, though embedded within the CAWI platform, was conducted asynchronously, meaning participants could provide their responses at different times, without interacting with one another in real time. This still captured in-depth responses and allowed participants to elaborate on their experiences, without requiring synchronous group discussions.

4. Cost-Effectiveness and Time Efficiency:

Embedding the VFG within the CAWI platform eliminated the need for separate meetings, saving time and resources while still allowing for rich data collection. This approach also made participation more convenient for the professionals, as they could take part in the survey at their own pace.

5. Streamlined Data Analysis:

The integration of both VFG and CAWI responses in one platform simplified the analysis process. Quantitative responses from closed-ended questions were analyzed statistically, while qualitative responses from the VFG were analyzed thematically. This provided a comprehensive understanding of the data, with statistical trends enriched by deeper qualitative insights.

#### 2.1.2. Setting

This study was conducted within healthcare environments across both public and private sectors in Italy, specifically in diagnostic laboratories and medical institutions where cytology practices are prevalent. The research focused on the integration of digital tools and AI into daily workflows, diagnostic procedures, and professional practices. The study aimed to assess how these technologies are being integrated into cytology practices within these healthcare settings.

#### 2.1.3. Participants

Participants in this study were selected based on their roles within cytology-related practices. The sample consisted of professionals from various fields, including the following:

- Laboratory Technicians: Professionals involved in the hands-on processes of cytological analysis, including sample preparation, processing, and initial evaluations.
- Medical Doctors: Specialists in pathology and diagnostic medicine, responsible for interpreting cytological results and guiding clinical decisions.
- Biologists: Professionals working within cytology laboratories, often providing diagnostic support and conducting research related to cell biology.
- Specialists in Health Professions of Diagnostic Technical Sciences: Professionals with specialized training in diagnostic imaging, medical laboratory technologies, and other technical sciences relevant to cytology practices.
- Participants were recruited from a diverse range of sources, including the following:
- Scientific societies specializing in cytology and pathology.
- Former students of university courses at Sapienza.
- Peer-to-peer recruitment methods through social media platforms (e.g., WhatsApp, LinkedIn) and professional networks.

The recruitment strategy ensured a diverse sample, capturing professionals from both urban and regional areas, with varying levels of experience and expertise. This variety allowed for a comprehensive understanding of the use and familiarity with digital cytology and AI tools across different roles and settings.

By combining these data collection methods and diverse participant sources, the study aimed to provide valuable insights into the current and potential future integration of digital cytology and AI within Italy’s healthcare system.

### 2.2. Data Collection Instruments

#### 2.2.1. Data Collection Tools: CAWI Instrument

The structured data collection for this study was facilitated through the use of Computer-Assisted Web Interviewing (CAWI). This tool was strategically employed to gather data from professionals potentially involved in discussions on cytology practices in healthcare settings. The use of CAWI ensured an efficient, flexible, and standardized approach to data collection, which was essential for gathering accurate responses from a diverse group of participants.

The CAWI tool was developed using Microsoft Forms, a platform integrated into the Office 365 (Version 2024) suite provided by the ISS. The selection of this platform was driven by several key factors, including its compliance with IT security regulations and institutional approval. This ensured that the data collection process adhered to the highest standards of data protection, mitigating concerns related to the use of external tools that would have required additional authorization and could have delayed the research process.

#### 2.2.2. Question Formats

The CAWI instrument employed various question formats to capture both quantitative and qualitative data, allowing for a comprehensive understanding of participants’ familiarity with and use of digital cytology and AI tools. These formats included the following:

- Single-choice questions: Used to gather straightforward, categorical data from participants, such as yes/no responses or selecting one option from a list.
- Multiple-choice questions: Allowed respondents to select more than one answer, providing a broader range of responses and allowing for nuanced data collection.
- Graded evaluation questions: These questions employed a six-level psychometric scale (ranging from 1 to 6) to assess participants’ familiarity and attitudes towards specific technologies or practices. The six levels enabled a more granular understanding of participants’ perceptions, which was vital for analyzing their familiarity with digital cytology and AI tools.
- Open-ended questions: These questions were selectively included to capture qualitative insights. Participants were given the opportunity to provide detailed, narrative responses that offered valuable context, particularly for understanding the factors influencing the adoption and use of AI in diagnostic workflows.
- Net Promoter Score (NPS) rating: in the CAWI, this question aimed to assess the participants’ willingness to recommend the procedure, with responses categorized into Promoters (9–10), Passives (7–8), and Detractors (0–6) and the final score derived by subtracting the percentage of Detractors from the percentage of Promoters.

#### 2.2.3. Ethical Integrity and Efficiency

The design and deployment of the CAWI tool were conducted with a focus on ensuring the efficiency, reliability, and ethical integrity of the research process. By leveraging the Microsoft Forms platform, the research team was able to ensure data security while streamlining the survey process.

#### 2.2.4. CAWI Methodology and Its Portability

The CAWI methodology is a versatile approach to data collection that facilitates the use of online surveys for research purposes. By incorporating a pseudocode to describe the survey flow and logic, this methodology can be easily adapted for various survey platforms such as Survey Monkey, Qualtrics, Lime Survey, and Google Forms.

The pseudocode, which outlines the survey’s structure and flow, is provided in Appendix A. It covers essential steps, including obtaining participation consent, gathering demographic information, assessing familiarity with digital cytology and AI, and identifying barriers to AI adoption. This pseudocode ensures that the core logic of the survey remains consistent, regardless of the platform being used.

This modular approach allows researchers to implement the same survey across multiple platforms without having to rewrite the survey’s logic for each tool. As a result, the survey design is both flexible and reproducible, supporting broader participant engagement and facilitating data collection with ease and consistency, no matter which survey tool is chosen.

### 2.3. Data Analysis Methodology

The data analysis for this study employed a combination of descriptive and inferential statistical methods to interpret the findings from the CAWI survey. These methods were selected to explore the relationships between various demographic factors, familiarity with digital cytology and AI tools, and the usage patterns of AI in diagnostic workflows. The aim was to provide a comprehensive understanding of how AI is integrated into the cytology field, focusing on a quantitative perspective.

#### 2.3.1. Descriptive Statistics

Descriptive statistics were utilized to summarize the core features of the collected data, providing an overall picture of the sample. This method allowed for the calculation of central tendencies and distributions, which were crucial for understanding the key characteristics of the population.

For categorical data such as sex, professional roles, and educational background, frequency distributions were calculated. This helped in understanding how participants were distributed across different demographic categories. Additionally, percentages were calculated for categorical responses, such as the use of AI tools in digital cytology workflows. This provided an insight into the proportion of respondents who were using these technologies, which was critical for analyzing adoption trends.

For continuous variables, such as the familiarity scores for digital cytology and AI tools, this study calculated measures of central tendency, including the mean, which indicated the average level of familiarity among participants. These measures helped provide a deeper understanding of how familiarity with these technologies varied across the samples.

#### 2.3.2. Inferential Statistics

To assess the statistical significance of observed patterns and relationships in the data, inferential statistical methods were employed. These methods helped to draw conclusions about the broader population based on the sample data.

The one-sample *t*-test was used to compare the mean scores for the graded evaluation questions on a six-level psychometric scale (ranging from 1 = minimum to 6 = maximum) against a predefined threshold. The threshold, calculated as (1 + 6)/2 = 3.5, served as the cutoff between low and high familiarity, with scores below 3.5 indicating low familiarity and scores above 3.5 indicating high familiarity. This test allowed the analysis to determine whether the average familiarity score for each technology was significantly different from the threshold. By doing so, it provided an insight into whether the overall sample was more familiar or unfamiliar with the technologies in question.

The Chi-square test was also employed to examine the relationship between categorical variables. This was particularly useful for analyzing responses to multiple-choice questions and single-choice questions. The Chi-square test was used to assess whether a higher level of familiarity with digital cytology was associated with an increased likelihood of using AI tools in professional practice. This test helped to identify whether there was a statistical relationship between two categorical variables, such as familiarity levels (categorized as low or high) and the usage of AI tools (yes/no). The results provided a clearer picture of whether familiarity with digital cytology influenced the adoption of AI tools.

#### 2.3.3. Thematic Analysis

In addition to the quantitative methods, a thematic analysis was conducted on the open-ended questions. This qualitative analysis allowed for the exploration of themes, patterns, and insights that emerged from participants’ written responses. The thematic analysis involved coding the responses to identify recurring ideas and categorizing these into broader themes. This process helped to provide context and deeper understanding of the factors influencing the use of AI tools in digital cytology and highlighted areas of concern or interest that were not captured through the structured questions.

#### 2.3.4. Data Cleaning and Preprocessing

Before conducting any statistical analyses, the data underwent a comprehensive cleaning process to ensure their integrity and reliability. This process involved handling missing data through appropriate methods such as imputation (where missing values were estimated) or exclusion (removing responses with insufficient data). Outlier detection was also performed to identify extreme values that could distort the results, ensuring the validity of the statistical tests.

For certain statistical tests, such as the *t*-test, the assumption of normality was checked. If necessary, data transformations were applied to normalize the distribution, allowing for more accurate results.

#### 2.3.5. Statistical Software Tools

All statistical analyses were performed using Microsoft (Redmond, Washington, DC, USA) Excel and IBM (Armonk, New York, NY, USA) SPSS Statistics 27.0. These tools were instrumental in efficiently processing the survey data, calculating the required statistical measures, and performing the inferential tests needed to draw conclusions from the data.

## 3. Results

The results are organized into four sections that align with the structure of the CAWI, which collected feedback from the VFG. Section 3.1, “Demographic Characteristics of Participants”, presents detailed demographic data about the respondents. Section 3.2, “Familiarity and Use of Advanced Technologies in Digital Cytology”, explores the participants’ familiarity with and use of technologies such as digital cytology and AI. Section 3.3, “Perceptions on the Integration of AI in Digital Cytology”, provides insights into how participants view the role of AI in digital cytology. Finally, Section 3.4, “Evaluation of Training and Resources for AI Integration”, discusses the adequacy of training and available resources for integrating AI tools into clinical practice. Each section provides an analysis corresponding to the relevant survey questions.

### 3.1. Demographic Characteristics of Participants

A total of 150 professionals participated in the study, contributing valuable insights into the demographic and professional composition of those involved in cytology practices across both public and private healthcare settings in Italy. The participants represented a diverse array of backgrounds, professional roles, and experience levels, which provides a broad perspective on the field of cytology.

#### 3.1.1. Sex Distribution

The sex distribution of the participants revealed a slight predominance of female professionals, who accounted for 52% (*n* = 78) of the total sample. Male participants represented 45% (*n* = 68) of the cohort, while a small percentage, 3% (*n* = 4), chose not to disclose their sex. This distribution reflects the broader trend seen in many healthcare professions, where women are often more represented in clinical and laboratory roles.

#### 3.1.2. Educational Background

The participants in the study came from a range of educational backgrounds, with the majority being professionals directly involved in diagnostic and cytological procedures. A breakdown of the educational qualifications is as follows:

- Laboratory technicians comprised 35% (*n* = 52) of the participants. This group is a significant component of the cytology workforce in both public and private laboratories, where they play a key role in sample processing and analysis.
- Medical doctors represented 25% (*n* = 38) of the sample, reflecting the involvement of physicians, particularly specialists in pathology and diagnostic medicine, who oversee and interpret cytology results.
- Biologists made up 20% (*n* = 30), another essential group within cytology laboratories, often engaged in diagnostic support and research.
- Specialists in health professions of diagnostic technical sciences accounted for 20% (*n* = 30). This group includes professionals trained specifically in diagnostic imaging, technical sciences, and medical laboratory technologies, who contribute to advanced diagnostic procedures in cytology.

**Figure 1:**
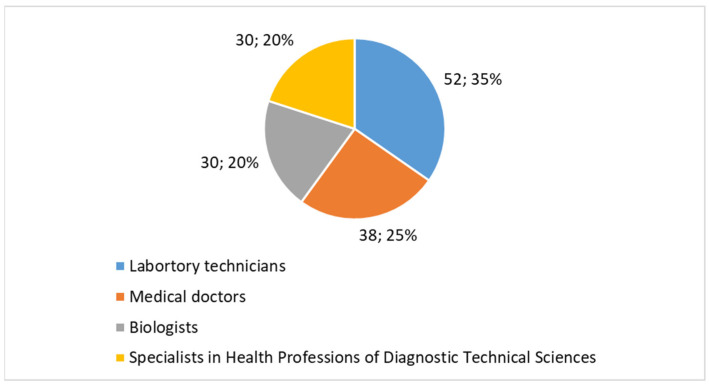
Proportions of participants.

The distribution (Figure 1) appears to be reasonably in line with the structure of cytology laboratories, where each professional group plays a distinct and crucial role. Laboratory technicians (35%) constitute the largest group, reflecting their essential role in sample processing and primary analysis. Medical doctors (25%) oversee diagnoses and provide clinical interpretation, while biologists and specialists in health professions of Diagnostic Technical Sciences (20% each) contribute to research, diagnostic support, and the integration of emerging technologies. This balanced representation ensures that insights into AI adoption encompass all critical aspects of digital cytology, from laboratory operations to clinical decision-making and technological advancement.

#### 3.1.3. Postgraduate Training in Cytology

Among the 150 participants, 44 had pursued a master’s or specialization in cytology. The most common titles included the following:

- A master’s in Cytopathology and Population Screening, which was the predominant specialization mentioned across the sample.
- Other notable programs included a specialization in Clinical Pathology and Cytology and master’s in Cervical Cytology and Population Screening. These qualifications are crucial for professionals working in both routine diagnostic and screening contexts, particularly in cervical cancer prevention and other related areas.

These demographic characteristics reflect a well-rounded and diverse group of professionals who play a critical role in the field of cytology. The combination of varied professional roles, postgraduate training, and years of experience contributes to a robust understanding of the practices and challenges within this area of healthcare.

### 3.2. Familiarity and Use of Advanced Technologies in Digital Cytology

This section explores the familiarity and usage of digital cytology and artificial intelligence (AI) in the diagnostic process. The familiarity with these technologies was assessed on a scale from 1 (very low familiarity) to 6 (very high familiarity), with a threshold of 3.5 to distinguish between low and high familiarity.

#### 3.2.1. Familiarity with Digital Cytology

When asked about their familiarity with digital cytology, the majority of participants reported a higher level of familiarity compared to AI.

The ratings for the familiarity in digital cytology were as follows, as reported in Table 1:

- Low familiarity (score 1−3): 50% (*n* = 75) of participants indicated low familiarity with digital cytology, with most scoring between 1 and 3, reflecting limited but existing exposure to digital platforms for cytological analysis.
- High familiarity (score 4−6): 50% (*n* = 75) reported higher familiarity with digital cytology, with most scores falling between 4 and 6, indicating a moderate to high level of comfort and experience with digital tools in cytology.

Average score: 3.9 (indicating a moderate to high level of familiarity with digital cytology). A one-sample *t*-test was conducted to compare the average familiarity score (M = 3.9) with the threshold of 3.5. The results were statistically significant (*t* (149) = 6.2, *p* < 0.001), indicating that the participants, on average, had a moderate to high level of familiarity with digital cytology.

#### 3.2.2. Familiarity with Artificial Intelligence (AI)

Familiarity with artificial intelligence was notably lower. A breakdown, reported in Table 2, is as follows:

- Low familiarity (score 1–3): A significant 75% (*n* = 113) of participants had low familiarity with AI, with scores predominantly in the lower range (1–3), suggesting limited or no exposure to AI technologies in clinical practice.
- High familiarity (score 4–6): Only 25% (*n* = 37) reported higher familiarity (scores 4–6), indicating that most participants have little to no practical experience or understanding of AI’s role in diagnostic workflows.

Average score: 2.2 (indicating a low level of familiarity with AI tools overall).

A one-sample *t*-test comparing the average familiarity score (M = 2.2) with the threshold of 3.5 showed a statistically significant difference (*t*(149) = −13.5, *p* < 0.001), suggesting that the majority of participants had a low level of familiarity with AI in the context of cytology.

#### 3.2.3. Use of AI Tools in Digital Cytology Workflow

When asked whether they had ever used AI tools in the digital cytology workflow, the responses revealed a clear gap in adoption (see Table 3):

- Yes: Only 35% (*n* = 52) of participants had used AI tools in their cytology practice, highlighting a moderate level of the integration of AI in diagnostic work, though still relatively low compared to the total number of participants.
- No: A large 65% (*n* = 98) of respondents had not used AI tools, indicating significant barriers to the adoption of AI technologies in the field.

The Chi-square test (χ^2^(1) = 14.11, *p* < 0.001) was statistically significant. This indicates that the adoption of AI tools in cytology practice was significantly lower than an equal 50−50 distribution.

#### 3.2.4. Types of AI Tools Used in Digital Cytology

Among the professionals who had used AI tools, the following applications (Table 4) were reported (multiple answers allowed):

- Automated Image Analysis: 50% (*n* = 26) used AI for automated image analysis to assist in identifying key cellular features and abnormalities.
- Support in Detecting Cellular Anomalies: 40% (*n* = 21) applied AI tools to help detect cellular anomalies, such as dysplasia or malignancy.
- Prediction of Diagnosis: 20% (*n* = 10) had used AI-based prediction tools for diagnosing conditions based on cytological findings.
- Other: 10% (*n* = 5) mentioned using additional AI applications not covered by the specific categories, suggesting some variability in the use of AI tools in cytology.

The most frequent AI application used in cytology was automated image analysis, with 50% (*n* = 26) of participants reporting its use.

### 3.3. Perceptions on the Integration of AI in Digital Cytology

This section explores how participants envision the role of artificial intelligence (AI) in enhancing digital cytology practices, including the types of AI tools that can be beneficial and the perceived barriers to their adoption.

#### 3.3.1. How Do You Think AI Can Be Useful in the Integration of Digital Cytology?

When asked about the potential benefits of AI in digital cytology, participants provided the following insights (multiple answers were allowed), also reported in Table 5:

- Improving Diagnostic Precision: 70% (*n* = 105) of participants believe AI will significantly enhance the precision of cytological diagnoses. They highlighted AI’s potential in reducing human error and providing consistent, reliable results.
- Reducing Analysis and Reporting Time: 55% (*n* = 82) feel that AI could dramatically reduce the time required for analysis and reporting, which could lead to faster decision-making and more efficient clinical workflows.
- Supporting the Detection of Hard-to-Identify Cellular Anomalies: 50% (*n* = 75) see AI as a powerful tool for identifying subtle or hard-to-detect anomalies, such as early-stage malignancies or rare cytological features that could be missed by human analysts.
- Optimizing Workflow and Sample Management: 45% (*n* = 68) believe AI could optimize workflows, automate repetitive tasks, and help manage cytology samples more effectively, leading to a more streamlined laboratory operation.
- Enabling Remote Access and Faster Consultations: 30% (*n* = 45) see AI facilitating the possibility of remote access to cytological data, enabling quicker consultations between experts and potentially improving the overall quality of care.
- Other: 5% (*n* = 8) provided additional suggestions, such as enhancing collaboration across multidisciplinary teams or supporting research in cytology.

The most frequent response to how AI could contribute to digital cytology was “Improving Diagnostic Precision”, with 70% (*n* = 105) of participants highlighting AI’s potential to reduce human error and provide more reliable results. This was followed by “Reducing Analysis and Reporting Time” (55%, *n* = 82) and “Supporting the Detection of Hard-to-Identify Cellular Anomalies” (50%, *n* = 75). These findings suggest that participants view AI’s role in enhancing diagnostic accuracy and efficiency as particularly valuable.

#### 3.3.2. How Do You Think You Will Contribute to the Integration of AI in the Digital Cytology Workflow?

Participants were asked how they saw their role in integrating AI into the cytology process. The responses (Table 6) indicated a proactive approach in working alongside AI technologies:

- Providing Training and Support for AI Tools: 50% (*n* = 75) indicated that they could contribute by offering training and guidance to colleagues, ensuring the proper usage and understanding of AI tools in everyday practice.
- Collaborating to Improve Diagnostic Accuracy: 30% (*n* = 45) believed their role would involve working closely with AI to refine its algorithms and improve its diagnostic capabilities, potentially offering feedback to developers.
- Adapting Workflows to Integrate AI Tools: 15% (*n* = 23) expressed a willingness to modify existing workflows to incorporate AI, adjusting processes to maximize the effectiveness of the new technologies.
- Other: 5% (*n* = 7) suggested other contributions, including being involved in the development of AI solutions specific to cytology needs or advocating for AI adoption in professional circles.

The most frequent response to the question about how participants would contribute to the integration of AI in the digital cytology workflow was “Providing Training and Support for AI Tools”, with 50% (*n* = 75) of participants indicating this as their expected contribution. A Chi-square test showed that this response was significantly more frequent than others (χ^2^(3) = 69.4, *p* <0.0001).

#### 3.3.3. Do You Think AI Will Be

Participants were asked to express their views on how AI will impact the role of professionals in digital cytology. The responses (Table 7) highlighted a cautious optimism for AI’s complementary role:

- Complementary to Human Work: 80% (*n* = 120) strongly believed that AI would complement human efforts rather than replace them, providing support and assistance in making more accurate and timely diagnoses.
- A Replacement for Human Work: 10% (*n* = 15) expressed concerns that AI might replace some human tasks, reflecting apprehension about job security and the future of clinical expertise.
- Unnecessary: 4% (*n* = 6) felt that AI would not be useful in digital cytology, potentially due to skepticism about or unfamiliarity with AI’s capabilities.
- Difficult to Integrate into the Workflow: 4% (*n* = 6) thought AI might be hard to incorporate into the existing workflow due to technical, procedural, or logistical challenges.
- Other: 2% (*n* = 3) indicated various views highlighting that AI could be a stepping stone to more automated systems or might lead to new forms of collaboration between clinicians and machines.

The Chi-square test for independence revealed a significant preference for the response “Complementary to Human Work” compared to the other categories (χ^2^(4) = 340.2, *p* < 0.001), indicating a highly significant difference from the expected equal distribution.

#### 3.3.4. What Barriers Do You Think Could Slow Down the Adoption of AI in Digital Cytology?

Participants were asked to identify the most significant obstacles that could hinder AI adoption in digital cytology. The following were the most commonly (see Table 8) cited barriers (multiple answers were allowed):

- Resistance to Change from Professionals: 60% (*n* = 90) mentioned that resistance from cytology professionals could be a major challenge. This could stem from concerns about job displacement, reluctance to adopt new technologies, or discomfort with AI’s role in decision-making.
- High Costs for Implementation and Maintenance: 55% (*n* = 82) noted that the financial cost of implementing and maintaining AI tools could be a significant obstacle, particularly in settings with limited budgets or financial resources.
- Concerns Over Image Quality and Scanning: 40% (*n* = 60) expressed worries about the quality of images produced by AI systems, fearing that low-quality scans or insufficient data might lead to inaccurate diagnoses.
- Difficulty Integrating with Existing Systems: 35% (*n* = 52) pointed out the potential technical difficulties in integrating AI tools with existing laboratory and hospital systems, especially if these systems are outdated or incompatible with new technology.
- Need for Continuous Staff Training and Updates: 30% (*n* = 45) highlighted the need for ongoing staff training to keep up with the evolving AI tools, which might require a sustained investment in education and professional development.
- Data Management and Privacy Concerns: 25% (*n* = 38) raised concerns over the security and privacy of patient data when using AI tools, especially in light of stringent data protection regulations such as the GDPR.
- Lack of Sufficient Clinical Evidence Supporting AI Effectiveness: 20% (*n* = 30) felt that the limited amount of clinical evidence demonstrating AI’s effectiveness in improving cytological diagnostics might slow its adoption, as professionals often require robust data to support new technologies.

The most frequently cited obstacle to AI adoption in digital cytology was “Resistance to Change from Professionals”, with 60% (*n* = 90) of participants noting that reluctance from cytology professionals could hinder AI integration. This was followed by “High Costs for Implementation and Maintenance” (55%, *n* = 82), and “Concerns Over Image Quality and Scanning” (40%, *n* = 60). These responses suggest that professional reluctance and financial barriers are major challenges to overcome for successful AI adoption in the field.

### 3.4. Evaluation of Training and Resources for AI Integration

In this section, participants were asked to evaluate the adequacy of training and resources available for integrating AI tools into their practice, provide optional feedback, and share their likelihood of recommending the survey.

#### 3.4.1. How Do You Evaluate the Adequacy of Training and Resources Available to Use AI Tools in Your Practice?

**Figure 2:**
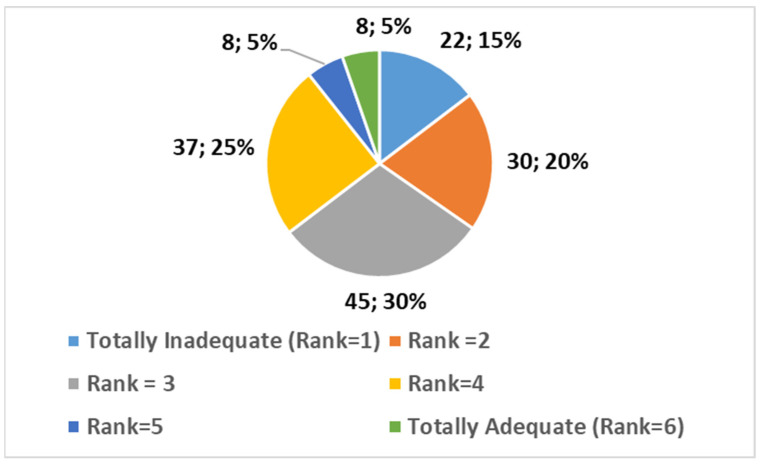
Perceived adequacy of training and resources.

Participants were asked to rate the adequacy of available training and resources on a scale from 1 (totally inadequate) to 6 (totally adequate). The distribution of responses (reported in Figure 2) was as follows:

- Totally Inadequate (1): 15% (*n* = 22);
- 2: 20% (*n* = 30);
- 3: 30% (*n* = 45);
- 4: 25% (*n* = 37);
- 5: 5% (*n* = 8);
- Totally Adequate (6): 5% (*n* = 8).

The average score was 3.02.

This suggests that while a significant portion of participants feel moderately confident in the resources and training available, there is still a gap that needs to be addressed to better equip professionals for AI integration.

A one-sample *t*-test comparing the average adequacy score (M = 3.02) with a neutral midpoint of 3,5 showed a statistically significant difference, suggesting that participants generally perceive the available training and resources as above neutral but not fully adequate (*p* < 0.001).

#### 3.4.2. Do You Have Any Comments or Observations?

Several participants provided feedback, highlighting both positive and constructive observations. A total of 34 comments were provided. After grouping similar themes, the comments are summarized in Table 9 below.

The feedback provided by participants reveals several important themes regarding AI training and its integration into digital cytology. The need for hands-on training was the most commonly mentioned theme, with 23.5% (*n* = 8) of participants indicating that they would benefit from more practical experience rather than just theoretical sessions. They emphasized the importance of real-world applications to better understand how AI tools function in clinical settings. Another significant point was the need for ongoing support and guidance, with 14.7% (*n* = 5) of respondents highlighting the importance of continuous assistance as AI tools rapidly evolve. They stressed that consistent updates and support would help practitioners stay current with new technologies and make the most out of their AI tools. Participants also expressed a desire for more comprehensive training materials, with 11.8% (*n* = 4) mentioning that the current resources do not provide in-depth coverage of AI tools and their full capabilities. These participants felt that more detailed content would allow for a better understanding of AI’s potential. Additionally, 8.8% (*n* = 3) mentioned the integration of AI in existing workflows as an important area. They suggested that training should include guidance on how to seamlessly incorporate AI tools into current work practices without disrupting existing processes. Concerns about AI’s reliability and usability were also raised by 8.8% (*n* = 3) of participants, who noted that some AI tools seemed unreliable or difficult to use effectively. This concern suggests the need for the further development of these tools to ensure they meet the standards of clinical practice. Finally, 5.9% (*n* = 2) of respondents mentioned the need for continuous updates on AI developments. These participants emphasized the importance of keeping up with the rapid pace of AI advancements to stay informed and competent in using these tools.

Overall, the feedback reflects the general satisfaction with the training but also indicates that there are several areas where improvements could be made. These include a greater focus on hands-on learning, better resources to understand the full potential of AI, and continuous support to help professionals adapt to new technologies as they evolve.

#### 3.4.3. How Likely Are You to Recommend This Survey to Others?

Participants were asked to rate the likelihood of recommending the survey and the procedure on a scale from 0 (not likely at all) to 10 (extremely likely). Based on the results, the Net Promoter Score (NPS) was calculated.

- Promoters (score 9–10): 60% (*n* = 90).
- Passives (score 7–8): 30% (*n* = 45).
- Detractors (score 0–6): 10% (*n* = 15).

The Net Promoter Score (NPS) is equal to %Promoters − %Detractors.

Using the provided data, the following is obtained: Promoters (score 9–10): = 60% and Detractors (score 0–6): 10%.

The Net Promoter Score (NPS): 50

A positive NPS indicates a favorable reception to the survey and procedure, with the majority of participants being Promoters.

The Chi-square test for goodness of fit confirmed that the distribution of responses was significantly different from the equal distribution across categories (χ^2^(2) = 57.0, *p* < 0.001), indicating that participants were significantly more likely to be Promoters of the survey.

## 4. Discussion

### 4.1. Summary and Highlights

Qualitative research based on focus groups with multiple professionals in the same field is crucial because it captures diverse perspectives, fosters in-depth discussions, and highlights shared challenges and opportunities. This approach enables the identification of trends, barriers, and best practices, enriching the understanding of complex topics. Additionally, it promotes interdisciplinary dialog, leading to more comprehensive and applicable insights for real-world implementation.

This study aimed to explore the integration of artificial intelligence AI into digital cytology workflows by surveying professionals involved in the field. Data were collected through the CAWI method, which was further enriched by feedback from a Virtual Focus Group. These two approaches provided a nuanced understanding of the perceptions, experiences, and challenges faced by professionals in the implementation and utilization of AI tools.

The results of the survey reveal a mixed level of familiarity with AI and digital cytology tools. While participants showed varying degrees of awareness, the integration of AI is still evolving within the practice. Many respondents recognized the potential of AI to improve diagnostic accuracy, streamline workflows, and assist in identifying complex cellular anomalies, which were seen as key advantages for digital cytology. However, barriers such as resistance to change, cost concerns, and doubts about the reliability and usability of AI tools emerged as significant hurdles to its broader adoption.

The feedback also highlighted the importance of proper training and continuous support to ensure successful AI integration. There was a notable call for more hands-on experience, practical examples, and ongoing guidance to navigate the rapid evolution of AI technologies. These findings underscore the need for tailored educational resources and robust systems to support professionals in adapting to the growing presence of AI within digital cytology.

Overall, the results emphasize that while AI has the potential to significantly transform digital cytology, its successful integration depends on addressing both technological and human factors. The feedback highlights the need for tailored educational initiatives, robust support systems, and strategies to overcome resistance, ensuring that professionals are adequately equipped to leverage AI’s full potential in practice. This study provides a valuable foundation for future efforts to enhance the role of AI in digital cytology and optimize its benefits for clinical outcomes.

### 4.2. Discussion of Added Value and Comparison to Existing Literature

#### Added Contribution of the Study

This study provides a structured analysis of AI integration in digital cytology, leveraging a Virtual Focus Group (VFG) and a Computer-Assisted Web Interview (CAWI) to gather diverse perspectives. The key added values of this approach are as follows:

First Added Value: The VFG offered a dynamic and interactive platform for discussing AI implementation, while the CAWI allowed for a broader reach, ensuring a comprehensive dataset. This approach’s added value lies in its ability to capture nuanced insights from professionals actively engaged in digital cytology workflows, providing a more detailed understanding of real-world challenges.

Second Added Value: Beyond the methodological advantages, the CAWI itself represents a significant asset, as it provides structured data collection and facilitates the systematic analysis of key trends within the field. This allows for a more rigorous and reliable assessment of AI adoption and its effects on digital cytology workflows.

Third Added Value: A major contribution of this study is its broad focus on different professional roles within the digital cytology ecosystem. Unlike previous surveys that primarily examined the perspectives of decision-makers and diagnosticians, this study actively includes laboratory technicians, IT specialists, and other key stakeholders. This holistic approach ensures a more representative understanding of the challenges and enablers of AI adoption, offering a well-rounded perspective that can inform future strategies.

Fourth Added Value: Finally, this research offers an initial demonstration of feasibility and provides guidance, outlining emerging trends that could inform future AI implementation strategies in digital cytology. This forward-looking aspect ensures that the findings contribute to the practical integration of AI in the field and sets the stage for future advancements. These findings align with and complement the prior research on AI adoption in digital cytology.

### 4.3. Comparing Consent and Acceptance in AI Integration in Digital Cytology: A Literature Context

The acceptance and consent of professionals involved in the integration of artificial intelligence (AI) into digital cytology workflows is a critical element in the broader process of adoption.

A PubMed search using the composite key “(digital cytology [Title/Abstract]) AND (artificial intelligence [Title/Abstract]) AND ((questionnaire [Title/Abstract]) OR (survey [Title/Abstract]))” identified only three relevant studies [22,23,41], underscoring the limited body of literature in this area regarding questionnaires and consensus initiatives. These studies, while providing valuable insights, highlight both the progress made and the significant challenges professionals face when integrating AI in digital cytology.

For example, Kim et al. [41] found that while whole slide imaging (WSI) is routine in surgical pathology (61%), cytology laboratories are still in the early stages of the digital transition, with only 46% scanning cytology slides. Despite growing interest in AI, only 13% of respondents in cytology were utilizing AI tools, compared to 16% in surgical pathology. These findings reveal the persistent challenges in AI adoption, such as concerns about scanning speed, image quality, and cost—issues that were also identified in our study. Our proposal extends the current literature by providing a structured analysis of AI integration through a Virtual Focus Group (VFG) and a Computer-Assisted Web Interview (CAWI), enabling the collection of diverse perspectives. Unlike Kim et al. [41], who focused primarily on adoption rates and barriers, our study captures a broader range of professionals involved in daily digital cytology operations. The VFG facilitated interactive discussions on AI implementation, while the CAWI gathered a comprehensive dataset, offering nuanced insights from a wider array of professionals. This includes feedback from laboratory technicians, IT specialists, and other key stakeholders, giving our study a more holistic approach and a deeper understanding of the enablers and challenges of AI adoption. For instance, laboratory technicians and IT specialists highlighted practical concerns, such as the integration of AI into workflows and infrastructure readiness—topics that were less explored in previous research.

The ASC’s Digital Cytology Task Force [22] examined the feasibility of integrating AI-driven whole slide scanning into cytology workflows. They identified benefits, such as enhanced efficiency and diagnostic accuracy, but also noted limitations, including the lack of real-world data and peer-reviewed studies on AI’s impact. Our study complements these findings by incorporating additional perspectives from those directly working with the technology and identifying emerging trends that could inform future AI implementation strategies.

Similarly, Kim et al. [23] emphasized the increasing adoption of digital cytology and AI but pointed out the need for more data to assess their full impact on cytology departments. Our study contributes by offering practical insights into the feasibility of AI integration and by highlighting the critical roles of various professionals in this adoption process.

Furthermore, our findings should be considered in the context of advancements in other fields, such as radiological imaging, where AI integration has progressed more rapidly due to a quicker adoption of digital technologies, particularly the DICOM standard in radiology [42] in comparison to digital pathology with the version DICOM WSI [43]. This comparison helps highlight how the pace of technological adoption and infrastructure standardization can influence the acceptance and consent processes in AI integration across different medical specialties.

A similar search in radiology on Pubmed—“((Artificial intelligence [Title/Abstract]) AND (Radiology [Title/Abstract])) AND ((Questionnaire [Title/Abstract]) OR (survey [Title/Abstract]))” [44] —yielded 181 studies, illustrating the broader focus on questionnaires and consensus initiatives within that field.

As highlighted in [45], questionnaires are vital in radiology for gathering insights on barriers to AI adoption, including infrastructure issues and image quality concerns, and for identifying opportunities for improvement. These insights help inform training programs, infrastructure updates, and policies to enhance technology integration.

Overall, while the use of questionnaires in radiology has proven critical in understanding AI challenges and opportunities, they can similarly be adapted and serve as a useful framework for advancing AI integration in digital cytology. By comparing the insights from our study with the literature, we can better navigate the challenges and design more effective strategies for AI adoption in digital cytology workflows.

### 4.4. Limitations of the Study

This *study* serves as an initial pilot experience, providing meaningful insights, particularly through a well-distributed sample of participants across different professional roles in digital cytology. However, it does not fully capture the numerical breadth of the sector at the national level. Achieving a more comprehensive representation would require broader initiatives, such as nationwide census efforts led by the Ministry of Health. If managed at the central administrative level with appropriate legislative support, the CAWI method could serve as a structured tool for conducting a more detailed and systematic census of professionals in the field.

Additionally, this study does not encompass all professional figures operating within hospital environments. While it includes laboratory technicians, pathologists, and IT specialists working in cytology laboratories, other key roles—such as economic and administrative professionals, who are likely experiencing or will experience significant impacts from AI integration in hospital management—are not well represented. Given the rapid evolution of AI technologies [12], particularly with the emergence of Large Language Models (LLMs) [46], it is crucial to continuously update the CAWI instrument to reflect these changes and to capture the diverse and evolving impacts of AI across different professional domains within cytopathology.

### 4.5. Perspectives

Despite these limitations, this study opens up multiple avenues for future research and practical applications. One promising area to monitor is the integration of the metaverse into digital cytology, which represents a significant leap toward a hyper-digitalized transformation of the field. The metaverse can combine physical and virtual realities to create immersive environments where healthcare professionals interact with AI-powered tools such as augmented reality, virtual reality, and blockchain technology. In the context of digital cytology, this can potentially revolutionize training for pathologists and cytologists by offering virtual hands-on experiences with AI tools for disease detection and classification. These virtual environments would not only enhance efficiency and accuracy but also broaden the scope for education and training in a dynamic, interactive setting [47].

Moreover, the metaverse could play a critical role in the development and prototyping of AI-driven medical practices, particularly in medical imaging. By facilitating virtual comparative scanning and data sharing, it can help enhance decision-making processes and patient care. AI-driven models and blockchain technology can ensure the secure management of patient data, offering a transparent and privacy-conscious environment in the metaverse [48]. As the metaverse continues to evolve, its integration with AI could significantly shape the future of digital cytology, pushing the boundaries of what is possible in both diagnosis and treatment [49].

Additionally, the importance of continuous training, highlighted by the findings from the CAWI, cannot be overstated. As noted in [50], AI-assisted training in digital cervical cytology has shown positive outcomes, such as improved diagnostic accuracy, reduced testing times, and enhanced consistency. AI tools have been demonstrated to increase sensitivity, specificity, and overall diagnostic performance. The majority of trainees in these studies reported significant improvements in their diagnostic skills, further emphasizing the value of incorporating AI into medical education. To fully capitalize on AI’s potential in digital cytology, it will be essential to continue adapting and refining training programs, ensuring that they remain aligned with the latest technological advancements.

## 5. Conclusions

This study explored the integration of AI in digital cytology using a combined approach of VFG and CAWI. These methods allowed for the collection of a broad range of perspectives from professionals across various fields, including laboratory technicians and IT specialists, providing an analysis of the challenges and opportunities in AI adoption. The results revealed a mixed level of familiarity with AI tools, with recognized advantages but also obstacles related to various factors. The study emphasizes the importance of targeted educational resources and continuous support to facilitate AI integration. The study offers an initial pilot experience with a reasonable distribution of participants across various professional roles. To achieve broader coverage, census initiatives led by the Ministry of Health could be valuable. Future perspectives include delegating the maintenance, updating, and specialization of AI tools to scientific societies, professional organizations, and industry associations, considering the rapid evolution of AI and increasing specialization in cytopathology.

## Figures and Tables

**Table 1:** Familiarity with digital cytology.

Familiarity Level	Score Range	Percentage	Number of Participants (*n*)	Description
Low familiarity	1–3	50%	75	Limited but existing exposure to digital cytology platforms.
High familiarity	4–6	50%	75	Moderate to high comfort and experience with digital tools.

**Table 2:** Familiarity with AI.

Familiarity Level	Score Range	Percentage	Number of Participants (*n*)	Description
Low familiarity	1−3	75%	113	Limited or no exposure to AI technologies in clinical practice.
High familiarity	4−6	25%	37	Little to no practical experience or understanding of AI’s role in diagnostic workflows.

**Table 3:** Adoption of AI.

AI Tool Usage	Percentage	Number of Participants (*n*)	Description
Yes	35%	52	Moderate level of AI integration in diagnostic work, though still relatively low.
No	65%	98	Significant barriers to AI adoption in cytology practice.

**Table 4:** Reported AI applications in cytology practice.

AI Application	Participants (*n*)	Percentage (%)
Automated Image Analysis	26	50%
Support in Detecting Cellular Anomalies	21	40%
Prediction of Diagnosis	10	20%
Other	5	10%

**Table 5:** Perceived benefits of AI integration in cytology.

AI Benefit Category	Percentage (%)	*n*	Description
Improving Diagnostic Precision	70%	105	AI is expected to enhance diagnostic accuracy, reduce human error, and provide consistent, reliable results.
Reducing Analysis and Reporting Time	55%	82	AI could significantly speed up analysis and reporting, leading to more efficient clinical workflows.
Supporting the Detection of Hard-to-Identify Cellular Anomalies	50%	75	AI can assist in identifying subtle anomalies, including early-stage malignancies and rare cytological features.
Optimizing Workflow and Sample Management	45%	68	AI could streamline laboratory operations by automating tasks and improving sample management.
Enabling Remote Access and Faster Consultations	30%	45	AI may facilitate remote access to cytological data, improving collaboration and consultation speed.
Other	5%	8	Additional suggestions included enhancing multidisciplinary collaboration and supporting research in cytology.

**Table 6:** Professionals’ perceived roles in AI integration in cytology.

Contribution to AI Integration	Percentage (%)	*n*	Description
Providing Training and Support for AI Tools	50%	75	Assisting colleagues with training and guidance to ensure proper use and understanding of AI tools.
Collaborating to Improve Diagnostic Accuracy	30%	45	Working alongside AI to refine algorithms and enhance diagnostic precision, potentially providing feedback to developers.
Adapting Workflows to Integrate AI Tools	15%	23	Modifying existing workflows to effectively incorporate AI technologies.
Other	5%	7	Involvement in AI development for cytology or advocating for AI adoption in professional settings.

**Table 7:** Participants’ views on AI’s impact on the role of professionals in digital cytology.

View on AI’s Impact	Percentage (%)	*n*	Description
Complementary to Human Work	80%	120	Strong belief that AI will complement human efforts by supporting more accurate and timely diagnoses.
A Replacement for Human Work	10%	15	Concerns that AI may replace some human tasks, reflecting apprehension about job security and the future of clinical expertise.
Unnecessary	4%	6	Belief that AI would not be useful in digital cytology, possibly due to skepticism about or unfamiliarity with its capabilities.
Difficult to Integrate into the Workflow	4%	6	View that AI might face challenges in integration due to technical, procedural, or logistical issues.
Other	2%	3	Various views suggesting AI might lead to more automated systems or new collaborations between clinicians and machines.

**Table 8:** Significant obstacles to AI adoption in digital cytology.

Obstacle	Percentage (%)	*n*	Description
Resistance to Change from Professionals	60%	90	Concerns about job displacement, reluctance to adopt new technologies, or discomfort with AI’s role in decision-making.
High Costs for Implementation and Maintenance	55%	82	Financial challenges in implementing and maintaining AI tools, particularly in resource-limited settings.
Concerns Over Image Quality and Scanning	40%	60	Worries about the quality of images produced by AI systems, potentially leading to inaccurate diagnoses due to low-quality scans or insufficient data.
Difficulty Integrating with Existing Systems	35%	52	Technical difficulties in integrating AI tools with existing laboratory and hospital systems, especially outdated or incompatible systems.
Need for Continuous Staff Training and Updates	30%	45	Ongoing staff training required to keep up with evolving AI tools, necessitating a sustained investment in education and professional development.
Data Management and Privacy Concerns	25%	38	Concerns about security and privacy of patient data when using AI tools, particularly with stringent data protection regulations like GDPR.
Lack of Sufficient Clinical Evidence Supporting AI Effectiveness	20%	30	Limited clinical evidence on AI’s effectiveness in improving cytological diagnostics, which may slow adoption due to need for robust supporting data.

**Table 9:** AI application categories and key contributions.

Comment Theme	Frequency
Need for hands-on training	8 (23.5%)
Ongoing support and guidance	5 (14.7%)
Desire for more comprehensive training materials	4 (11.8%)
Integration of AI in existing workflows	3 (8.8%)
Concerns about AI’s reliability and usability	3 (8.8%)
Need for continuous updates on new AI developments	2 (5.9%)
General satisfaction	3 (8.8%)
Desire for more clarity on AI’s role	1 (2.9%)
Concerns about AI replacing jobs	1 (2.9%)
Lack of technical support	1 (2.9%)
Need for clearer guidelines on AI’s implementation	1 (2.9%)
Preference for more practical experience	1 (2.9%)
Concerns about AI’s reliability and usability	3 (8.8%)
Need for continuous updates on new AI developments	2 (5.9%)

## Data Availability

Data are contained within the article.

## Appendix A. Pseudocode for the CAWI Survey

To provide a clear and structured outline of the survey process, a pseudocode is included below. This pseudocode outlines the sequence of actions and decisions involved in the survey, allowing for a systematic approach to data collection. It serves as a guide for replicating the survey process on similar platforms, such as Google Forms or SurveyMonkey, and illustrates the logical flow of the survey from participation consent to final submission.

The pseudocode is designed to be portable and adaptable for use with various survey tools, ensuring that the survey’s design can be implemented consistently across different platforms. The following pseudocode outlines the steps and responses, highlighting key decision points and mandatory responses required for survey completion.

**Step 1:** Participation Consent and Basic Demographics.
  (1) Participation Consent.
    ○ Participation in this survey is voluntary, and data will be collected anonymously in compliance with applicable regulations. Consent is required to participate.
    ○ Mandatory Response: Single choice.
      ■ Yes.
      ■ No (exit the survey).
  (2) Do you work in a cytology laboratory?
    ○ Mandatory Response: Single choice.
      ■ Yes.
      ■ No (exit the survey).
  (3) What is your age?
    ○ Mandatory Response: Single line text.
      ■ Enter your age in years.
      ■ The value must be a number.
  (4) What is your sex?
    ○ Mandatory Response: Single choice.
      ■ Male.
      ■ Female.
      ■ Prefer not to disclose.

**Step 2:** Educational Background and Work Experience--.
  (5) What is your educational background?
    ○ Mandatory Response: Single choice.
      ■ Laboratory Technician.
      ■ Doctor.
      ■ Biologist.
      ■ Specialist in diagnostic technical health sciences.
      ■ Other.
  (6) Do you have a Master’s degree or specialization focused on cytology?
    ○ Mandatory Response: Single choice.
      ■ Yes.
      ■ No (jump to question 8).
  (7) Please enter the title of your Master’s or specialization with a focus on cytology.
    ○ Mandatory Response: Single line text.
      ■ Enter your answer.
  (8) How familiar are you with digital cytology?
    ○ Mandatory Response: Rating scale (1 = minimum; 6 = maximum).
  (9) How familiar are you with artificial intelligence (AI)?
    ○ Mandatory Response: Rating scale (1 = minimum; 6 = maximum).
  (10) Have you ever used AI tools in the digital cytology workflow?
    ○ Mandatory Response: Single choice.
      ■ Yes.
      ■ No (jump to question 12).
  (11) What type of AI tools have you used?
    ○ Mandatory Response: Multiple choice (select all that apply).
      ■ Automatic image analysis.
      ■ Support in detecting cellular abnormalities.
      ■ Diagnosis prediction.
      ■ Other.

**Step 3:** AI in Digital Cytology Integration--.
  (12) How do you think artificial intelligence can be useful in the integration of digital cytology?
    ○ Mandatory Response: Multiple choice (select all that apply).
      ■ Improving diagnostic accuracy.
      ■ Reducing analysis and reporting times.
      ■ Supporting the detection of hard-to-identify cellular abnormalities.
      ■ Optimizing workflow and sample management.
      ■ Enabling remote access and faster consultation.
      ■ Other.
  (13) How do you think you can contribute to the integration of AI into digital cytology workflows?
    ○ Mandatory Response: Single choice.
      ■ Providing training and support in the use of AI tools.
      ■ Collaborating to improve diagnostic accuracy.
      ■ Adapting work processes to integrate AI tools.
      ■ Other.
  (14) Do you think artificial intelligence will be:
    ○ Mandatory Response: Single choice.
      ■ Complementary to human work.
      ■ A replacement for human work.
      ■ Useless.
      ■ Difficult to integrate into my workflow.
      ■ Other.

**Step 4:** Barriers to AI Adoption--.
  (15) What barriers do you think could slow down the adoption of artificial intelligence in digital cytology?
    ○ Mandatory Response: Multiple choice (select all that apply).
      ■ Resistance to change from professionals.
      ■ High costs for implementation and maintenance.
      ■ Concerns regarding image quality and scanning.
      ■ Difficulty integrating with existing systems.
      ■ Need for continuous staff training and updates.
      ■ Data management and privacy issues.
      ■ Lack of clinical evidence supporting AI effectiveness.
      ■ Other.

**Step 5:** Training and Resources
  (16) How would you rate the adequacy of the training and resources available to use AI tools in your practice?
    ○ Mandatory Response: Rating scale (1 = totally inadequate; 6 = totally adequate).

**Step 6:** Open Comments
  (17) Add any comments or observations.
    - Text paragraph.
    - Enter your answer.

**Step 7:** Survey Recommendation
  (18) How likely are you to recommend our survey?
    ○ Mandatory Response: Net Promoter Score (NPS) rating.
